# CKD in Sri Lanka - A Prevalence Study

**DOI:** 10.1016/j.ekir.2025.11.034

**Published:** 2025-12-05

**Authors:** Rajitha A. Abeysekera, Helen G. Healy, S. Samita, Sampath Tennakoon, Sakunthala Jayasinghe, Nuwan D. Wickramasinghe, Indika Gawarammana, Navin Gamage, Vishwa Hemakeerthi, Farook Rafsanjani, T.M.W.V. Tennakoon, Sachini N. Palliyaguru, Wendy E. Hoy

**Affiliations:** 1 School of Medicine, University of Queensland, Brisbane, Queensland, Australia; 2 Department of Medicine, Faculty of Medicine, University of Peradeniya, Sri Lanka; 3 Centre for Education Research Training in Kidney Disease, Faculty of Medicine, University of Peradeniya, Sri Lanka; 4 Kidney Health Service, Royal Brisbane and Women’s Hospital, Brisbane, Queensland, Australia; 5 Conjoint Internal Medicine Laboratory, Chemical Pathology, University of Queensland, Brisbane, Queensland, Australia; 6 Department of Crop Science, Faculty of Agriculture, University of Peradeniya, Sri Lanka; 7 Department of Community Medicine, Faculty of Medicine, University of Peradeniya, Sri Lanka; 8 Department of Pathology, Faculty of Medicine, University of Peradeniya, Sri Lanka; 9 Faculty of Medicine and Allied Health Sciences, Rajarata University of Sri Lanka

**Keywords:** chronic kidney disease, prevalence, Sri Lanka

## Introduction

Chronic kidney disease (CKD) is common and increasing worldwide, disproportionately affecting people in developing countries.^1^ Most South Asian countries are low or middle income with large but poorly documented burdens of kidney disease and limited access to health care, kidney replacement therapy, and skilled workforce.^2^ The prevalence of CKD in the region is estimated to be 14% with marked variability within and between countries.^3^ Two systematic reviews^3^^,^^4^ identified the prevalence of CKD at 10.2% to 16% in India, 14% to 17.3% in Bangladesh, 12% to 21.2% in Pakistan and 6% to 10.6% in Nepal. No Sri Lankan data were available. The prevalence of CKD is projected to increase in the region as the number of people with diabetes mellitus (DM) and hypertension (HTN) increases.

Similar health burdens are present in Sri Lanka, with rising prevalences of kidney risk factors such as DM (prevalence: 23%^5^), HTN (prevalence: 28.1%–51.3%^6^), aging population and lifestyle shifts to the metabolic phenotype. The country is ill-prepared to respond, lacking accurate data to support evidence based nationwide health care planning and funding.

This prevalence study was conducted to address the current significant gap in our knowledge of the scale of CKD beyond CKD of unknown etiology (CKDu) in Sri Lanka. This study aimed to estimate the prevalence of CKD and enumerate underlying associated risk factors. The study was cross-sectional, using a multistage, stratified, random-cluster-sampling design with proportional allocation (Supplementary Methods; Supplementary Tables S1–S3). It was conducted in Kandy district (Supplementary Figure S1), a CKDu non-endemic region, between August 2022 and August 2023. Representative groups of adults aged ≥ 18 years from urban, rural, and estate (i.e., plantation areas) residential sectors were sampled.

## Results

Characteristics of 1843 participants recruited into the study (Supplementary Figure S2) are summarized in Supplementary Table S4. They reflected the national distribution of place of residence, with 13.9% (*n* = 257) urban, 78.2% (*n* = 1442) rural, and 7.8% (*n* = 144) estate sectors. Median age was 56 (interquartile range: 30–81) years, with 45.7% aged 40 to 59 years and 38.7% aged 60 to 79 years. Females (58.5%) and people of Sinhalese ethnicity (80.3%) predominated; patterns repeated across the 3 residential sectors. Of the study population, 26.3% (*n* = 485) met the criteria for DM and 60.9% (*n* = 1122) for HTN.

The crude prevalence of CKD was 14.4% (*n* = 265) (Table 1). Crude prevalence standardized by age groups was 10.7% (95% confidence interval [CI]: 10.6%–10.8%). The majority (86.8%, *n* = 230) were with early CKD, grades 1 to 3A, and 13.2% (*n* = 35) were with CKD grades 3B to 5. Crude prevalences varied substantially across residential sectors as follows: 12.5% (95% CI: 8.9%–17.1%) in the urban sector, 15.4% (95% CI 13.6%–17.3%) in the rural sector, and 7.6% (95% CI: 4.2%–13.2%) in the estate sector.

**Table 1:** Distribution of CKD in the study sample by diagnostic criterion and the residential sector

Diagnostic criterion	Sector			
	Total	Urban	Rural	Estate
Self-reported CKD				
Yes	59 (3.2)	14 (5.5)	44 (3.1)	1 (0.7)
No	1584 (86.4)	202 (79.8)	1265 (88.1)	117 (81.2)
Not sure	190 (10.4)	37 (14.6)	127 (8.8)	26 (18.1)
CKD - by eGFR (ml/min/1.73 m^2^)				
≤ 60	124 (6.7)	23 (8.9)	92 (6.4)	9 (6.2)
> 60	1719 (93.3)	234 (91.0)	1350 (93.6)	135 (93.7)
CKD - by albuminuria	*n* = 1825	*n* = 248	*n* = 1434	*n* = 143
Positive	180 (9.9)	15 (6.1)	161 (11.2)	4 (2.8)
Dipstick positive	48	9	38	1
Urine ACR positive	132	6	123	3
Negative	1645 (90.1)	233 (93.9)	1273 (88.8)	139 (97.2)
CKD status				
Low eGFR alone	85 (32.1)	17 (53.1)	61 (27.5)	7 (63.6)
Albuminuria alone	141 (53.2)	9 (28.1)	130 (58.6)	2 (18.2)
Combination^a^	39 (14.7)	6 (18.7)	31 (13.9)	2 (18.2)
Overall CKD (eGFR ± albuminuria)				
CKD	265 (14.4)	32 (12.5)	222 (15.4)	11 (7.6)
No CKD	1578 (85.6)	225 (87.5)	1220 (84.6)	133 (92.4)
CKD stage				
Grade 1	94 (35.5)	5 (15.6)	87 (39.2)	2 (18.2)
Grade 2	47 (17.7)	4 (12.5)	43 (19.3)	0 (0)
Grade 3A	89 (33.6)	13 (40.6)	70 (31.5)	6 (54.5)
Grade 3B	22 (8.3)	5 (15.6)	15 (6.8)	2 (18.2)
Grade 4	8 (3.0)	3 (9.4)	4 (1.8)	1 (9.1)
Grade 5	5 (1.9)	2 (6.2)	3 (1.3)	0 (0.0)
Total	1843	257 (13.9)	1442 (78.2)	144 (7.8)

ACR, albumin-to-creatinine ratio; CKD, chronic kidney disease; eGFR, estimated glomerular filtration rate.

a Combination – presence of both low glomerular filtration rate and albuminuria.

Participants with CKD were significantly older; median age was 65 (interquartile range: 26–89) years compared with 55 (interquartile range: 18–92) years in participants without CKD (Table 2). The proportion of participants with CKD increased with age, with prevalence at 6.1% (95% CI: 3.7%–9.9%) in the 18 to 39 years age group, 9.1% (95% CI: 7.4%–11.3%) in the 40 to 59 years age group, 22.4% (95% CI: 19.5%–25.6%) in the 60 to 79 years age group, and 30.9% (95% CI: 18.8%–46.5%) in the ≥ 80 years age group. CKD was present in 25.6% (*n* = 124) of participants with DM (*n* = 485) and 19% (*n* = 213) with HTN (*n* = 1122).

**Table 2:** Comparison of number of participants with CKD versus no CKD

Characteristic	CKD	No CKD	χ^2^ value	*P*-value
Sector			7.3	0.026
Urban	32 (12.5)	225 (87.5)		
Rural	222 (15.4)	1220 (84.6)		
Estate	11 (7.6)	133 (92.4)		
Age (median; range)	65 (26-89)	55 (18-92)		
18–39 yrs	15 (6.1)	230 (93.9)	79.4	< 0.001
40–59 yrs	77 (9.1)	766 (90.9)		
60–79 yrs	160 (22.4)	553 (77.6)		
≥ 80 yrs	13 (30.9)	29 (69.1)		
Sex			9.6	0.002
Female	132 (49.8)	946 (59.9)		
Male	133 (50.2)	632 (40.1)		
Ethnicity			8.7	0.07
Sinhala	218 (82.3)	1260 (79.8)		
SL Tamil	15 (5.7)	170 (10.8)		
SL Moor	30 (11.3)	138 (8.7)		
Indian Tamil	1 (0.4)	8 (0.5)		
Burgher	1 (0.4)	2 (0.1)		
Marital status			9.6	0.086
Married	246 (92.8)	1421 (90.1)		
Unmarried	4 (1.5)	88 (5.6)		
Widowed	10 (3.8)	44 (2.8)		
Divorced	0 (0.0)	3 (0.2)		
Separated	0 (0.0)	1 (0.1)		
Unknown	5 (1.9)	21 (1.3)		
Education level			7.9	0.24
Not attended school	12 (4.5)	46 (2.9)		
Primary^a^	56 (21.1)	261 (16.5)		
Secondary^b^	118 (44.5)	737 (46.7)		
Up to A/L	49 (18.5)	348 (22.1)		
Higher education	9 (3.4)	49 (3.1)		
Other	17 (6.4)	92 (5.8)		
Unknown	4 (1.5)	45 (2.8)		
Employment			22.2	< 0.001
Employed	59 (22.3)	515 (32.6)		
Unemployed	154 (58.1)	863 (54.7)		
Pensioner	47 (17.7)	159 (10.1)		
Student	0 (0.0)	12 (0.8)		
Unknown	5 (1.9)	29 (1.8)		
Diabetes mellitus	*n* = 1674		59.02	<0.001
Yes	124 (49.2)	361 (25.4)		
No	128 (50.8)	1061 (74.6)		
Hypertension			51.2	<0.01
Yes	213 (81.3)	909 (58.1)		
No	49 (18.7)	657 (41.9)		
IHD			8.19	0.004
Yes	23 (8.7)	71 (4.5)		
No	242 (91.3)	1507 (95.5)		
Stroke			0.95	0.33
Yes	6 (2.3)	23 (1.5)		
No	259 (97.7)	1555 (98.5)		
Dyslipidemia			7.30	0.007
Yes	65 (24.5)	277 (17.5)		
No	200 (75.5)	1301 (82.4)		
Kidney stones			4.42	0.036
Yes	15 (5.7)	49 (3.1)		
No	250 (94.3)	1529 (96.9)		
BMI			0.81	0.85
Underweight	18 (6.9)	94 (6.0)		
Normal	111 (43.0)	666 (42.9)		
Overweight	75 (29.1)	486 (31.3)		
Obese	54 (20.9)	307 (19.8)		
Total	265	1578		

A/L, advanced level examination; BMI, body mass index; IHD, ischemic heart disease.

a Primary – up to year 5.

b Secondary – up to ordinary level examination.

Residential sector, age, sex, employment status, DM, HTN, ischemic heart disease, dyslipidemia, and kidney stones were significantly associated with CKD by univariant analyses (*P* < 0.05) (Table 2). However, multiple logistic regression analyzing the independence of these variables identified only age, sex, DM, and HTN as significantly associated with CKD (*P* < 0.05) (Supplementary Table S5). The proportion of participants with CKD increased with age with odds ratio (OR) of 2.0 (95% CI: 1.1–3.6) at 60 to 79 years and OR of 2.9 (95% CI: 1.1–7.2) at age ≥ 80 years compared with the referent age group of 18 to 39 years. Males, patients with DM, and those with HTN had higher risks of CKD with OR of 1.6 (95% CI: 1.1–2.2), OR of 2.4 (95% CI: 1.8–3.3), and OR of 2.3 (95% CI: 1.6–3.2), respectively.

Only a third (37.8%, *n* = 691) of participants self-reported that they knew risk factors for kidney disease (Supplementary Table S6). Asked as open-ended questions, 41.7% (*n* = 764) said they did not know the causes of CKD, only 7.5% (*n* = 139) and 2.7% (*n* = 51) identified DM and HTN, respectively as causes of CKD but 31.6% (*n* = 580) cited polluted water. When asked as a closed questions 43% (*n* = 783) agreed DM and 33.8% (*n* = 629) agreed HTN were causes of CKD.

## Discussion

This study provides the first accurate estimates of CKD prevalence among adults in a CKDu non-endemic region in Sri Lanka. We identified age-standardized prevalence of 10.7% (95% CI: 10.6%–10.8%). The study identified increasing age, as well as presence of DM and HTN as significant factors associated with CKD, a risk profile shared with the populations of developed economies. Males had 1.6 higher prevalence of CKD than females. Most studies report a higher prevalence among females.^7^ However, a male predominance, as in our study, was reported in Thailand and Japan.^8^

Contrary to our expectations, the prevalence of CKD in the rural sector was similar to the urban sector (12.5% vs. 15.4%). Sri Lanka’s sociodemographic landscape is changing. Civil governance continues to separate the sectors administratively; however, lifestyle risk factors show similar patterns. The study identified low awareness of the common CKD risk factors in a country with a successful public health program in CKDu. Sri Lanka is among global leaders in responding to CKDu. The current challenge is shaping a national policy to respond to the threat of CKD beyond the CKDu pattern.

The study demonstrates the value of screening high-risk groups, even in non-CKDu endemic regions, for early interventions. Early CKD grades 1 to 3A were found in 86.8% of participants with CKD, where evidence-based interventions are proven to slow or halt progression of disease. Government kidney prevention programs that incorporate national initiatives for risk factors such as DM and HTN are likely to produce responses that better fit the health needs of the population.

This study demonstrates the feasibility and the reality of research in a low and middle-income country. We encountered community trust or distrust, difficulties of access to some participant groups, and challenges of a national economy under strain. The study had limitations. First, the 18 to 39 years age group was underrepresented. This group was absent from their electoral households during the study periods, travelling for education and work. To correct for this, CKD prevalence was standardized for the group’s age structure in Kandy district and crude prevalence was adjusted down to 10.7%. Second, estimated glomerular filtration rate equations are not validated in the Sri Lankan population; and the sensitivity, that is, over- or under- estimation of glomerular filtration rate is unknown. Third, study measurements, particularly kidney function and HTN, were made on a single day as opposed to repeated measurements specified in guidelines,^9^ a remedial amendment of the study protocol in response to unforeseen national economic contraction.

The strengths of the study include the standardized sampling technique, which provides accurate estimates across participant groups representative of the national population. Participants with any abnormality benefited by referral to local health care providers. Given the representativeness of the sample, these results can be cautiously extrapolated to other settings with similar age structures and sectoral distributions.

In conclusion, this study provides the first accurate estimates of CKD prevalence in a representative population of adult Sri Lankans in a CKDu non-endemic region of the country. We identified a standardized CKD prevalence of 10.7% (95% CI: 10.6–10.8) and a CKD risk profile comparable to other countries in South Asia and globally. The burden of CKD in Sri Lanka is expected to increase, and global projections are therefore relevant for Sri Lanka’s health system.

## Disclosure

All the authors declared no competing interests.

## Supplementary Material

Supplementary File (PDF) **Supplemental Methods. Supplementary References. Figure S1.** Twenty Divisional Secretariate divisions, Kandy district, Sri Lanka. **Figure S2.** Flow chart of recruitment of study participants. **Table S1.** Estimates of sample size by participant age group and residential sector in Kandy. **Table S2.** Data frame – Total population in Kandy district by age category and residential sector. **Table S3.** Data sampling frame-number of sample units by residential sector. **Table S4.** Characteristics of study participants by residential sector. **Table S5.** Relationship of CKD and risk factors – multiple logistic regression analysis. **Table S6.** Responses to awareness questions on kidney diseases. STROBE Statement.

## Footnotes

Supplementary File (PDF)

**Supplementary Methods.**

**Supplementary References.**
**Figure S1.** Twenty Divisional Secretariate divisions, Kandy district, Sri Lanka. **Figure S2.** Flow chart of recruitment of study participants. **Table S1.** Estimates of sample size by participant age group and residential sector in Kandy. **Table S2.** Data frame – Total population in Kandy district by age category and residential sector. **Table S3.** Data sampling frame-number of sample units by residential sector. **Table S4.** Characteristics of study participants by residential sector. **Table S5.** Relationship of CKD and risk factors – multiple logistic regression analysis. **Table S6.** Responses to awareness questions on kidney diseases. STROBE Statement.
