# Supplementary material for: CKD in Sri Lanka - A Prevalence Study
Source: Kidney Int Rep. 2025 Dec 5;11(2):103710. doi: 10.1016/j.ekir.2025.11.034 (PMC12805018; doi:10.1016/j.ekir.2025.11.034)
Supplement: Supplementary File (PDF) — Supplemental Methods. Supplementary References. Figure S1. Twenty Divisional Secretariate divisions, Kandy district, Sri Lanka. Figure S2. Flow chart of recruitment of study participants. Table S1. Estimates of sample size by participant age group and residential sector in Kandy. Table S2. Data frame – Total population in Kandy district by age category and residential sector. Table S3. Data sampling frame-number of sample units by residential sector. Table S4. Characteristics of study participants by residential sector. Table S5. Relationship of CKD and risk factors – multiple logistic regression analysis. Table S6. Responses to awareness questions on kidney diseases. STROBE Statement. [file mmc1.pdf]

Supplementary Material

Supplementary Methods

Supplementary References

Supplementary Figures S1-2

Supplementary Tables S1-5

STROBE checklist

Supplementary Methods

Ethical approval

The study was approved by the Ethics Review Committee of the Faculty of Medicine, University of Peradeniya, Sri Lanka (No:2022/EC/01). The study was supported by the University Research Grant URG/2022/48/M, University of Peradeniya, Sri Lanka. Administrative clearances were obtained from the District Secretariate (DS) office, Provincial Director of Health Services of Central Province and Regional Director of Health Services in Kandy, Sri Lanka. Informed, written consent was obtained prior to data collection.

Study setting

The study was conducted in Kandy district of Sri Lanka between August 2022 and August 2023. Kandy district's population is representative of that of Sri Lanka and is the third most densely populated district with 1,375,382 people at the last national census report[S1]. Kandy district is administratively organized into 20 Divisional Secretariats (DS) and these into 1,187 Grama Niladhari (GN) divisions[S1] (**Supplementary Figure S1**). GN divisions are further

organized into urban, rural and estate (i.e. plantation areas) sectors based on economic development and living standards of people.

Sampling method

The sample size was calculated by a multi-stage, stratified, random-cluster-sampling design with proportional allocation[S2]. In the absence of prevalence estimates of CKD in Sri Lanka, sample size estimation assumed a CKD prevalence of 20% on the basis of prevalence estimates reported in other South Asian countries[3,4], a confidence level of 95% and precision of 0.03. As the study design involved cluster sampling, a design effect was considered, and a conservative estimate of 2.5 was used due to the absence of published data on intraclass correlation coefficients from a similar population. The study sample size was estimated at 1,784 participants (**Supplementary Table S1**).

Recruitment plan

Adults ≥18 years of age and on the residential list of Kandy District were eligible for recruitment. Stratification was done by residential sector (urban, rural and estate) and age-category (18-39, 40-59, 60-79 and ≥80 years) as these factors have been identified to have an impact on the CKD prevalence. The probability proportional to size (PPS) allocation method was applied to the Kandy district population (**Supplementary Table S2**) to select the number of sample units in each strata (**Supplementary Table S1**). Representation by residential sectors of urban, rural or estate was achieved by iterative rounds of PPS, firstly from the DS divisions (DSD) in Kandy District. Ten (10) out of the total twenty (20) DS divisions were randomly selected (urban = 2 DSD, rural = 6 DSD, estate = 2 DSD) (**Supplementary Table S3**). Randomly selected DS divisions then underwent PPS allocations of their GN divisions (Supplementary Table 3). The third round of PPS randomly selected households from the most updated residents lists in the GN divisions identified in the previous round. A household was

considered a sampling (study) unit. Only one individual in a relevant age category was selected from each household. The selection process was repeated until the sample size was achieved for each of the age categories (**Supplementary Table S3**). Adults ≥18 years of age with a history of acute kidney injury (AKI) within the preceding 3 months were not eligible for recruitment.

Recruitment of participants

A total of 2,152 participants were screened, of whom 295 did not consent and 14 excluded either due to insufficient biological samples for analysis or missing data. The final sample size was 1,843 participants (**Supplementary Figure S2**).

Data collection

Data was sourced from interviewer-administered questionnaires and clinical data logged at the time of field visits as well as the participants' clinical records (**Supplementary Table S4**).

Blood pressure (BP) was measured using a standard digital BP monitor (Omron HEM-7120, Omron Healthcare Co. Ltd, Japan). BP was measured according to a standard protocol where the participant was seated and rested for 5 minutes. Three readings were taken 1 minute apart with the average of the last two values being recorded. Height was measured using a measuring tape attached to the wall to the nearest 0.1cm. Body weight (BW) was measured using an electronic weighing scale according to the manufacturer's protocol.

Biological samples

Blood and urine samples were collected. 5ml of blood was collected for serum creatinine and fasting plasma glucose (FPG) measurements into a plain tube and a sugar (NaF/K oxalate) tube respectively. Serum and plasma, respectively, were separated and stored at -80°C. Serum

creatinine was analyzed using the enzymatic assay validated to the isotope dilution mass spectrometry (IDMS) standards. FPG was analyzed by the glucose oxidase method. Both tests were performed in an automated analyzer calibrated to quality control standards. Estimated glomerular filtration rate (eGFR) was calculated using the Chronic Kidney Disease Epidemiology Collaboration (CKD-EPI) equation. Participants were given containers to collect urine on the morning of the study visit. An aliquot of 10ml of the early morning void urine was reserved and urinary protein dipstick testing was performed on the remaining sample onsite. The reserved urine aliquots of participants with dipstick negative, trace or 1+ albuminuria were stored in a cooler in an ice box at 4°C. These samples were sent to the clinical medical laboratories of Department of Medicine, Faculty of Medicine, University of Peradeniya and Nephrology Unit, National Hospital Kandy `and urine albumin:creatinine ratio (UACR) was measured within 24-hours.

Clinical data

Participants were categorized as having hypertension if systolic blood pressure (SBP) ≥140mmHg and/or diastolic blood pressure (DBP) ≥90mmHg, defined according to the International Society of Hypertension 2020 guidelines[S3], or if taking any antihypertensive medication.

Participants were categorized as having diabetes mellitus (DM) if already diagnosed with diabetes, on treatment for diabetes or whose biological sample tested a FPG ≥ 126mg/dL, as defined by the American Diabetes Association 2024 guidelines[S4]. Pre-diabetes was defined as FPG of 100 – 125 mg/dL and FPG <100mg/dL as no diabetes.

Participant body mass was categorized as underweight <18.5, normal 18.5 – 24.9, overweight 25 – 29.9 or obese ≥30 kg/m^2, according to WHO recommendations[S5].

Participants were categorized as CKD if already diagnosed with CKD or whose GFR was estimated <60ml/min/1.73m^2 and/or urine tested positive for albumin[9]. CKD was categorized into grades according to the KDIGO CKD Consensus Statement. Albuminuria was recorded as positive if urine dipstick was 2+ or more or if the urine albumin:creatinine ratio (ACR) >30mg/g.

Follow up

All participants were given the results of their clinical measurements and investigations via mail. Participants with high BP, high FPG, high BMI, impaired serum creatinine and/or albuminuria results were referred to the nearest public hospital or medical practitioner for further evaluation.

Statistical analysis

Categorial variables were summarized as percentages or proportions, continuous variables distributed normally were summarized as means and standard deviations (SD) and continuous variables not normally distributed with median and interquartile ranges (IQR). The prevalence of CKD was calculated as a point estimate and 95% confidence interval (CI). To account for the underrepresentation of the 18-39 years age group in the study sample, crude prevalence was age-standardized extrapolating from the age distribution of the originally calculated sample proportions that were representative of the population of the Kandy District.

The relationships of the possible individual risk factors of sector, age, sex, ethnicity, educational level, employment, comorbidities (diabetes, hypertension, ischaemic heart disease (IHD), stroke, dyslipidaemia, kidney stones and BMI) and CKD were explored by chi-square testing in univariant statistical models (**Supplementary Table S5**). Variables with p-value of <0.05 were progressed to multivariate logistic regression models to identify the independence

of the association of significant risk factors with CKD. A p-value of <0.05 was considered significant. STATA 14.1 statistical software was used for data analysis.

Supplementary References

S1. Department of Census &Statistics MoPPaEA, Sri Lanka. *Census of Population and Housing 2012*. Statistics DoC; 2012.

S2. Cochran WG. Sampling techniques. John Wiley & Sons; 1977.

S3. Unger T, Borghi C, Charchar F, et al. 2020 International Society of Hypertension Global Hypertension Practice Guidelines. *Hypertension*. Jun 2020;75(6):1334-1357. doi:10.1161/hypertensionaha.120.15026

S4. American Diabetes Association. (2024). *Standards of care in diabetes—2024. Diabetes Care, 47*(Supplement 1), S1–S312.

S5. Physical status: the use and interpretation of anthropometry. Report of a WHO Expert Committee. *World Health Organ Tech Rep Ser*. 1995;854:1-452.

Supplementary Figures

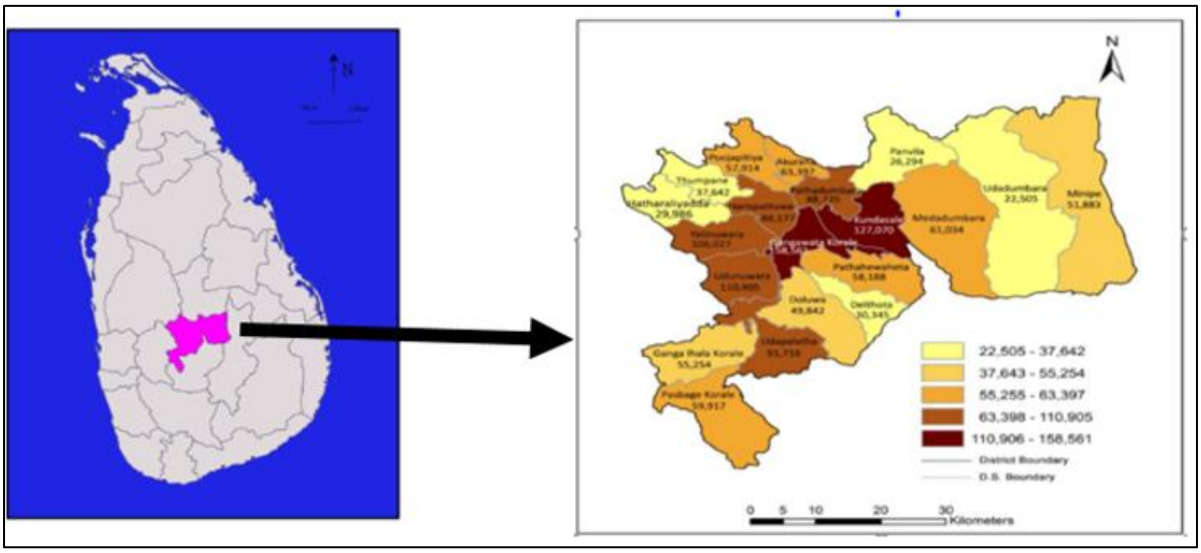

Supplementary Figure S1. Twenty Divisional Secretariate divisions, Kandy District, Sri Lanka

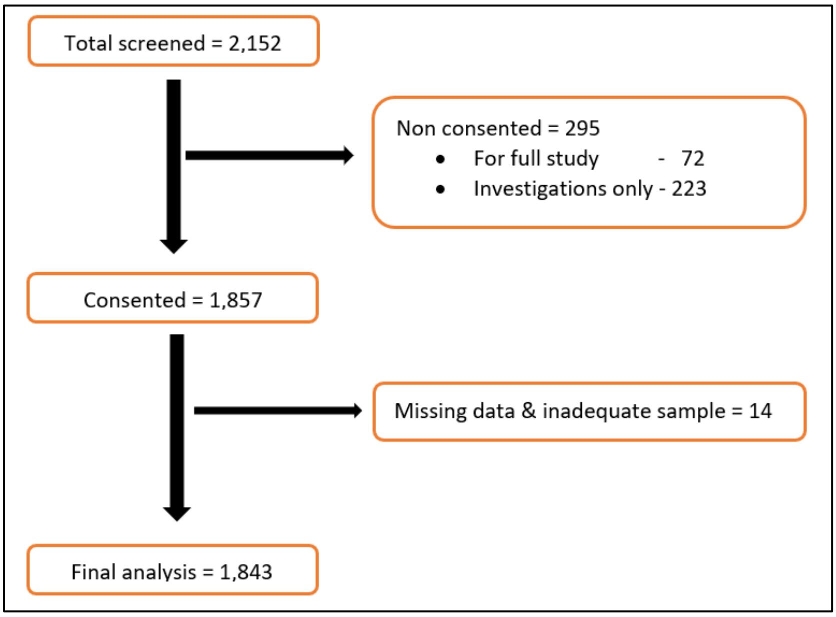

Supplementary Figure S2. Flow chart of recruitment of study participants

Supplementary Tables

Supplementary Table S1: Estimates of sample size by participant age group and residential sector in Kandy District

		Population aged ≥18 years		Estimated study sample	
		n	%	n	%
Total		914,449		1784	
Age group (years)					
	18 - 39	387,207	42.3	754	42.3
	40 - 59	341,209	37.3	665	37.3
	60 - 79	165,702	18.1	324	18.1
	≥80	20,331	2.2	41	2.2
Residential sector in Kandy District					
	Urban	133,600	14.6	261	14.6
	Rural	724,154	79.2	1411	79.1
	Estate	56,695	6.2	112	6.2

source: census of population & Housing 2012, Ministry of Policy & Planning and Economic Affairs[1]

Supplementary Table S2: Data frame – Total population in Kandy District by age category and residential sector

DS division	GN divisions	Total population	Male	Female	age groups				Totals
	n	n	n	n	18 – 39	40-59	60-79	≥80	
Thumpane	67	37642	18215	19427	10395	9556	5093	722	25766
Pujapitiya	67	57914	27327	30587	15625	14325	7096	883	37929
Akurana	35	63397	29940	33457	17206	13717	5586	611	37120
Pathadumbara	52	88725	41920	46805	24428	22450	10513	1271	58662
Panvila	14	26294	12213	14081	7158	6133	3320	318	16929
Udadumbara	63	22505	11040	11465	6392	5786	2713	356	15247
Minipe	48	51883	25468	26415	16025	12684	5016	647	34372
Medadumbara	93	61034	28852	32182	17014	15187	7203	927	40331
Kundasale	80	127070	60589	66481	38389	31716	14837	2037	86979
Kandy Four Gravets & Gangawata Korale	64	158561	76284	82277	47015	40464	19832	2807	110118
Harispattuwa	84	88177	41767	46410	24562	22547	10878	1437	59424
Hatharaliyadda	57	29986	14242	15744	7961	7849	4143	604	20557
Yatinuwara	95	106027	50921	55106	29134	27434	14025	1797	72390
Udunuwara	124	110905	53554	57351	31228	27247	13080	1617	73172
Doluwa	33	49842	24407	25435	13902	12058	6010	605	32575
Pathahewaheta	73	58188	28030	30158	16267	14837	7199	906	39209
Deltota	29	30345	14179	16166	7901	6905	3618	284	18708
Udapalatha	49	91716	42716	49000	24909	22227	11302	1074	59512
Ganga Ihala Korale	31	55254	26539	28715	15690	13634	6929	747	37000
Pasbage Korale	29	59917	27588	32329	16006	14453	7309	681	38449
TOTAL					387207	341209	165702	20331	914449

Census of population & Housing 2012, Ministry of Policy & Planning and Economic affairs[1]

Supplementary Table S3: Data sampling frame-number of sample units by residential sector

	Total sample	Urban	Rural	Estate
number(%)	1843	257 (13.9)	1442 (78.2)	144 (7.8)
**DS Division**				
Gangawata korale		206 (80.2)		
Yatinuwara		51 (19.8)		
Dethota			107 (7.4)	
Ganga ihala korale			163 (11.3)	
Harispaththuwa			344 (23.9)	
Minipe			194 (13.4)	
Pathahewaheta			214 (14.8)	
Udunuwara			420 (29.1)	
Doluwa				46 (31.9)
Panvila				98 (68.1)
**GN division**				
Bahirawakanda		26 (10.12)		
Heerassagala		27 (10.51)		
Ilukwatta		32 (12.45)		
Kadugannawa		19 (7.39)		
Lewella		26 (10.12)		
Suduhumpola west		30 (11.67)		
Watapuluwa		26 (10.12)		
Watapuluwa south		23 (8.95)		
Wattaranthanna		20 (7.78)		
Welata		28 (10.89)		
Bulathwelkandura			64 (4.44)	
Dambagahawela			63 (4.37)	
Galpaya			30 (2.08)	
Gampolawela			73 (5.06)	
Ganguldeniya			103 (7.14)	
Godamunna west			68 (4.72)	
Hamangoda north			81 (5.62)	
Katakubura			98 (6.80)	
Kiriwawala			67 (4.65)	
Kotagaloluwa			56 (3.88)	
Narangaskubura			89 (6.17)	
Nilawala			78 (5.41)	
Rabbegamuwa			96 (6.66)	
Rathmale			95 (6.59)	
Sinharagama			68 (4.72)	
Suduwella			55 (3.81)	
Uggala			79 (5.48)	
Wadiyagoda			52 (3.61)	
Waragolla			67 (4.65)	
Watakedeniya			60 (4.16)	
Pupuressa				46 (31.94)
Thawalanthenna				98 (68.06)

Supplementary Table S4: Characteristics of study participants by residential sector

Characteristic		Sector		
	Total	Urban	Rural	Estate
	n (%)	n (%)	n (%)	n (%)
Age (years)				
median (IQR)	56 (30–81)	57 (22–89)	57 (19-87)	48 (28-76)
18-39	245 (13.3)	22 (8.6)	185 (12.8)	38 (26.4)
40-59	843 (45.7)	125 (48.6)	656 (45.5)	62 (43.1)
60-79	713 (38.7)	105 (40.8)	567 (39.3)	41 (28.5)
≥80	42 (2.3)	5 (1.9)	34 (2.4)	3 (2.1)
Sex				
Female	1,078 (58.5)	134 (52.1)	858 (59.5)	86 (59.7)
Male	765 (41.5)	123 (47.9)	584 (40.5)	58 (40.3)
Ethnicity				
Sinhala	1,478 (80.3)	233 (90.3)	1,232 (85.4)	14 (9.7)
Sri Lankan Tamil	185 (10.0)	12 (4.3)	45 (3.1)	129 (89.6)
Sri Lankan Moor	168 (9.1)	14 (5.4)	154 (10.7)	0 (0.0)
Indian Tamil	9 (0.5)	0 (0)	8 (0.5)	1 (0.7)
Burgher	3 (0.2)	0 (0)	3 (0.2)	0 (0.0)
Marital status				
Married	1,667 (90.5)	234 (91.1)	1,305 (90.5)	128 (88.9)
Unmarried	92 (4.9)	19 (7.4)	66 (4.5)	7 (4.9)
Widowed	54 (2.9)	3 (1.2)	42 (2.9)	9 (6.2)
Divorced	3 (0.2)	0 (0.0)	3 (0.2)	0 (0.0)
Separated	1 (0.1)	0 (0.0)	1 (0.1)	0 (0.0)
Unknown	26 (1.4)	1 (0.4)	25 (1.7)	0 (0.0)
Education level				
Not attended school	58 (3.2)	1 (0.4)	35 (2.4)	22 (15.3)
Primary[†]	317 (17.2)	21 (8.2)	233 (16.2)	63 (43.7)
Secondary[*]	855 (46.4)	99 (38.5)	713 (49.4)	43 (29.9)
Up to A/L[±]	397 (21.5)	92 (35.8)	294 (20.4)	11 (7.6)
Higher education	58 (3.2)	32 (12.4)	26 (1.8)	0 (0.0)
Other	109 (5.9)	8 (3.1)	100 (6.9)	1 (0.7)
Unknown	49 (2.7)	4 (1.6)	41 (2.8)	4 (2.8)
Employment				
Employed	574 (31.1)	101 (39.3)	369 (25.6)	104 (72.2)
unpaid employment	1,017 (55.2)	83 (31.9)	920 (63.8)	15 (10.4)
Pensioner	206 (11.2)	68 (26.5)	118 (8.2)	20 (13.9)
Student	12 (0.6)	2 (0.8)	9 (0.6)	1 (0.7)
Unknown	34 (1.8)	4 (1.6)	26 (1.8)	4 (2.8)

Comorbidities				
IHD††	94 (5.1)	26 (10.1)	64 (4.4)	4 (2.8)
Stroke	29 (1.6)	6 (2.3)	22 (1.5)	1 (0.7)
Dyslipidaemia	342(18.6)	69 (26.8)	268 (18.6)	5 (3.5)
Kidney Stones	64 (3.5)	0 (0)	64 (4.4)	0 (0)
Diabetes mellitus				
Previously diagnosed	415 (22.5)	94 (36.6)	315 (21.8)	6 (4.2)
Newly diagnosed	70 (3.8)	1 (0.3)	62 (4.3)	7 (4.8)
Total	485 (26.3)	95 (36.9)	377 (26.1)	13 (9.0)
Hypertension				
Previously diagnosed	545 (29.6)	82 (31.9)	439 (30.4)	24 (16.7)
Newly diagnosed	577 (31.3)	81(31.5)	442 (30.7)	54 (37.5)
Total	1,122 (60.9)	163 (63.4)	881 (61.1)	78 (54.2)
BMI (n=1811)				
Median (IQR)	23.6 (20.8-26.7)	24.8 (22.2-27.7)	23.6 (21.1-26.7)	20.2 (18.5-23.6)
Underweight	112 (6.1)	6 (2.3)	81 (5.6)	25 (17.4)
Normal	777 (42.2)	80 (31.1)	615 (42.6)	82 (56.9)
Overweight	561 (30.4)	89 (34.6)	446 (30.9)	26 (18.1)
Obese	361 (19.6)	66 (25.7)	285 (19.8)	10 (6.9)
TOTAL	1843	257 (13.9)	1442 (78.2)	144 (7.8)

†Primary – up to year 5, *secondary – up to ordinary level examination, ±A/L - advanced level examination, †IHD - ischaemic heart disease

Supplementary Table S5. Relationship of CKD and risk factors – multiple logistic regression analysis

	**OR (95% CI)**	**P value**
Sector	0.85 (0.5-1.2)	0.37
Age category	0.6 (0.4-0.7)	<0.01
18 – 39*	1.0	
40 - 59	0.8 (0.4-1.6)	0.67
60 - 79	2.0 (1.1-3.6)	0.02
≥80	2.9 (1.1-7.2)	0.02
Sex		
Female*	1.0	<0.01
Male	1.6 (1.1-2.2)	
Employment status	1.1 (0.9-1.2)	0.06
Diabetes	2.4 (1.8-3.3)	<0.001
Hypertension	2.3 (1.6-3.2)	<0.001
IHD	1.1 (0.6-1.9)	0.68
Dyslipidaemia	0.9 (0.6-1.3)	0.73

*Reference group

Supplementary Table S6: Responses to awareness questions on kidney diseases

Question		Responses		
	n (%)	Yes	No	Don't know
Do you get screening done for kidney disease?	1824	271 (14.9)	1547 (84.8)	6 (0.3)
Do you know risk factors for kidney disease?	1829	691 (37.8)	834 (45.6)	304 (16.6)
What are causes of kidney disease?				
Do not know	764 (41.7)			
Polluted water	580 (31.6)			
Medicine	195 (10.6)			
Diabetes mellitus	139 (7.5)			
Hypertension	51 (2.7)			
Dehydration	140 (7.6)			
Pesticides	8 (0.1)			
Other	100 (0.5)			
Can diabetes cause kidney disease?	1821	783 (43)	804 (44.1)	234 (12.8)
Can high blood pressure cause kidney disease?	1830	619 (33.8)	922 (50.4)	289 (15.8)

*Polluted water-collectively includes answers related to water contamination by pollutants and toxins

STROBE Checklist

STROBE Statement—Checklist of items that should be included in reports of ***cross-sectional studies***

	Item No	Recommendation	Page
No			
Title and abstract	1	(a) Indicate the study's design with a commonly used term in the title or the abstract	4
Introduction			
Background/rationale	2	Explain the scientific background and rationale for the investigation being reported	4
**Objectives**			
Methods			
Study design	4	Present key elements of study design early in the paper	S
Setting	5	Describe the setting, locations, and relevant dates, including periods of recruitment, exposure, follow-up, and data collection	S1
Participants	6	(a) Give the eligibility criteria, and the sources and methods of selection of participants	S2
Variables	7	Clearly define all outcomes, exposures, predictors, potential confounders, and effect modifiers. Give diagnostic criteria, if applicable	S4
Data sources/ measurement	8*	For each variable of interest, give sources of data and details of methods of assessment (measurement). Describe comparability of assessment methods if there is more than one group	S3-4
Bias	9	Describe any efforts to address potential sources of bias	NA
Study size	10	Explain how the study size was arrived at	S2
Quantitative variables	11	Explain how quantitative variables were handled in the analyses. If applicable, describe which groupings were chosen and why	S5
Statistical methods	12	(a) Describe all statistical methods, including those used to control for confounding	S5
		(b) Describe any methods used to examine subgroups and interactions	NA
		(c) Explain how missing data were addressed	NA
		(d) If applicable, describe analytical methods taking account of sampling strategy	NA
Results			

Participants	13*	(a) Report numbers of individuals at each stage of study—eg numbers potentially eligible, examined for eligibility, confirmed eligible, included in the study, completing follow-up, and analysed	5
		(b) Give reasons for non-participation at each stage	NA
		(c) Consider use of a flow diagram	S3
Descriptive data	14*	(a) Give characteristics of study participants (eg demographic, clinical, social) and information on exposures and potential confounders	5
		(b) Indicate number of participants with missing data for each variable of interest	5
Outcome data	15*	Report numbers of outcome events or summary measures	5
Main results	16	(a) Give unadjusted estimates and, if applicable, confounder-adjusted estimates and their precision (eg, 95% confidence interval). Make clear which confounders were adjusted for and why they were included	5
		(b) Report category boundaries when continuous variables were categorized	5
		(c) If relevant, consider translating estimates of relative risk into absolute risk for a meaningful time period	
**Other analyses**			
Discussion			6-7
Key results	18	Summarise key results with reference to study objectives	6
Limitations	19	Discuss limitations of the study, taking into account sources of potential bias or imprecision. Discuss both direction and magnitude of any potential bias	7
Interpretation	20	Give a cautious overall interpretation of results considering objectives, limitations, multiplicity of analyses, results from similar studies, and other relevant evidence	8
**Generalisability**			
Other information			

*Give information separately for exposed and unexposed groups.

Note: An Explanation and Elaboration article discusses each checklist item and gives methodological background and published examples of transparent reporting. The STROBE checklist is best used in conjunction with this article (freely available on the Web sites of PLoS Medicine at http://www.plosmedicine.org/, Annals of Internal Medicine at http://www.annals.org/, and Epidemiology at http://www.epidem.com/). Information on the STROBE Initiative is available at www.strobe-statement.org.
